# 1109. A multicenter, observational study to compare the effectiveness of Ceftazidime-Avibactam versus Ceftolozane-Tazobactam for multidrug-resistant *Pseudomonas aeruginosa* infections in the United States (CACTUS)

**DOI:** 10.1093/ofid/ofad500.082

**Published:** 2023-11-27

**Authors:** Ryan K Shields, Lilian M Abbo, Renee Ackley, Samuel L Aitken, Benjamin Albrecht, Ahmed Babiker, Renzo Cifuentes, Kimberly C Claeys, Kathryn DeSear, Jason C Gallagher, Eric Gregory, Emily L Heil, Carissa Hickey, Megan Klatt, Ellen G Kline, Ryan C Kubat, Wesley D Kufel, Jae Hyoung Lee, Ahmi Lim, Theresa Lingg, Conan MacDougall, Amy Mathers, Erin K McCreary, William J Moore, Shannon Olson, Jessica Oxer, Jeffrey C Pearson, Christine Pham, Christopher Polk, Michael J Satlin, Sarah W Satola, Sunish Shah, Yash B Solanki, Pranita Tamma, Ana Vega, Venugopalan Veena, Michael Veve, Walaiporn Wangchinda, Lucy S Witt, Janet Wu, jason M Pogue

**Affiliations:** University of Pittsburgh, Pittsburgh, PA; University of Miami Miller School of Medicine, Miami Transplant Institute and Jackson Health System, Miami, FL; Atrium Health, Charlotte, North Carolina; Michigan Medicine, Ann Arbor, Michigan; Emory University, Atlanta, Georgia; Emory University School of Medicine, Atlanta, GA; University of Miami Miller School of Medicine, Miami, Florida; University of Maryland Baltimore, Baltimore, MD; University of Florida Health Shands Hospital, Gainesville, Florida; Temple University School of Pharmacy, Philadelphia, PA; The University of Kansas Health System, Kansas City, MO; University of Maryland School of Pharmacy, Baltimore, MD; Temple University School of Pharmacy, Philadelphia, PA; The University of Kansas Health System, Kansas City, MO; University of Pittsburgh, Pittsburgh, PA; University of Kansas, Kansas City, Kansas; Binghamton University School of Pharmacy and Pharmaceutical Sciences, Binghamton, NY; Johns Hopkins, Baltimore, Maryland; University of California, San Francisco (UCSF) Medical Center, San Francisco, California; University of Pittsburgh, Pittsburgh, PA; University of California San Francisco, San Francisco, CA; University of Virginia, Charlottesville, VA; UPMC, Pittsburgh, PA; Northwestern Medicine, Chicago, Illinois; Sinai-Grace Hospital Detroit Medical Center, Detroit, Michigan; Weill Cornell Medicine, New York, New York; Brigham and Women's Hospital, Boston, Massachusetts; University of California, Los Angeles; David School of Medicine/University of California, Los Angeles, Los Angeles, California; Atrium Health, Charlotte, North Carolina; Weill Cornell Medicine, New York, New York; Emory University School of Medicine, Division of Infectious Diseases, Atlanta, Georgia; Antibiotic Management Program, UPMC Presbyterian Hospital, Pittsburgh, PA, Pittsburgh, Pennsylvania; Temple University School of Pharmacy, Philadelphia, PA; Johns Hopkins School of Medicine, Baltimore, MD; Jackson Memorial Hospital, Miami, Florida; University of Florida, College of Pharmacy, Gainesville, Florida; Henry Ford Health, Detroit, Michigan; University of Michigan College of Pharmacy, Ann Arbor, MI; Emory University, Atlanta, Georgia; Cleveland Clinic, Cleveland, Ohio; University of Michigan, College of Pharmacy, Ann Arbor, Michigan

## Abstract

**Background:**

Ceftolozane-tazobactam (CT) and ceftazidime-avibactam (CZA) are front-line agents for treatment of multidrug-resistant (MDR) *Pseudomonas aeruginosa*; however, real-world comparative-effectiveness data are lacking.

**Methods:**

CACTUS is a retrospective, matched, multicenter study to compare the efficacy of CT and CZA among patients with bacteremia or pneumonia due to MDR *P. aeruginosa.* CT and CZA patients were matched 1:1 within each study site by the presence/absence of septic shock/severe sepsis, infection site, and time to treatment initiation. The primary outcome was clinical success at day 30 defined as survival, resolution of signs/symptoms with the intended treatment course, and absence of recurrent infections. Patients with cystic fibrosis or COVID-19 infection within 90 days were excluded.

**Results:**

234 patients were included from 20 sites. Patient demographics, severity of illness, infection types, and treatment durations were similar for patients treated with CT or CZA (**Table 1**). The overall median age was 61 years, 61% were male, and the median Charlson score was 5. At study drug initiation, 77% of patients were in the ICU, 67% received mechanical ventilation and the median SOFA score was 7. 79% of patients were treated for pneumonia; 72% of which occurred in ventilated patients. The median time from index culture to treatment initiation was 72 hours in both groups; CT patients were more likely to receive a prolonged infusion of ≥3 hours (36% vs 19%; *P*=0.005). Clinical success occurred in 62% and 55% of patients receiving CT and CZA, respectively (*P*=0.35; **Table 1**). Corresponding rates of success for pneumonia were 63% and 52%, respectively (*P*=0.13; **Figure 1**). All-cause, 30-day mortality rate was 20% and 19%, respectively. Microbiologic failures, recurrent infections, and development of resistance within 90 days were similar between groups. Time to a composite endpoint of recurrent infection or death within 90 days was similar between groups in the overall analysis and the subgroup of patients with pneumonia (**Figure 2**).

**Figure:**
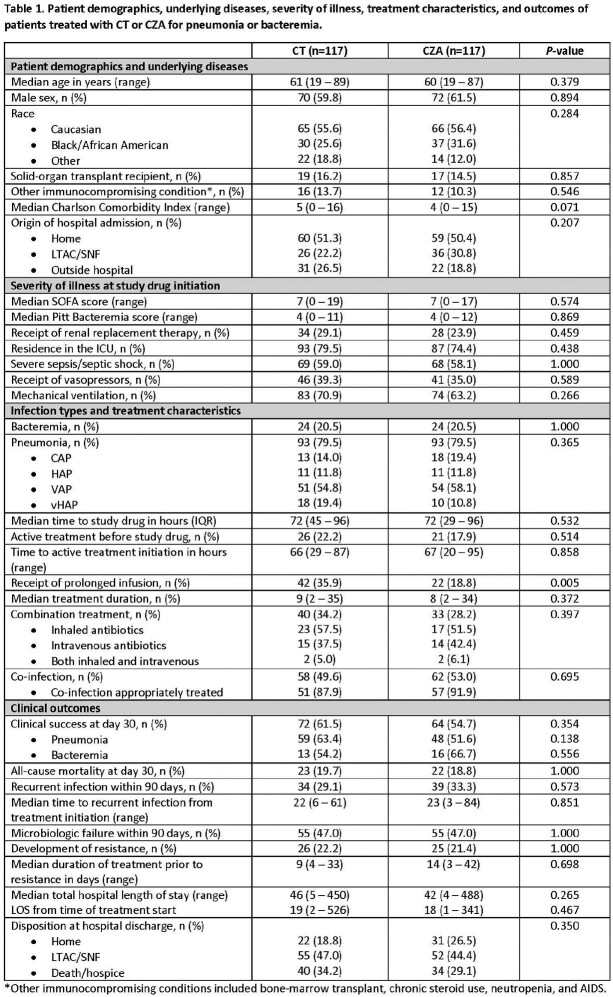

**Figure:**
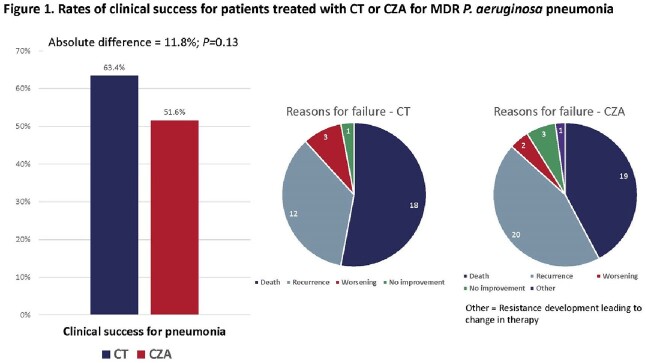

**Figure:**
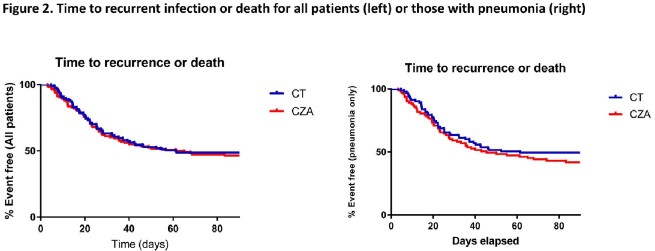

**Conclusion:**

In this interim analysis of the CACTUS study, patients treated with CT and CZA had similar clinical outcomes. We plan to continue enrollment up to 420 patients to detect if any differences exist in the efficacy of CT and CZA for MDR *P. aeruginosa* infections.

**Disclosures:**

**Ryan K. Shields, PharmD, MS**, Allergan: Advisor/Consultant|Cidara: Advisor/Consultant|Entasis: Advisor/Consultant|GSK: Advisor/Consultant|Melinta: Advisor/Consultant|Melinta: Grant/Research Support|Menarini: Advisor/Consultant|Merck: Advisor/Consultant|Merck: Grant/Research Support|Pfizer: Advisor/Consultant|Roche: Grant/Research Support|Shionogi: Advisor/Consultant|Shionogi: Grant/Research Support|Utility: Advisor/Consultant|Venatorx: Advisor/Consultant|Venatorx: Grant/Research Support **Lilian M. Abbo, MD, MBA**, Ferring: Advisor/Consultant|Pfizer: Advisor/Consultant|Regeneron: Grant/Research Support|Shionogi: Advisor/Consultant **Ahmed Babiker, MBBS**, Roche: Advisor/Consultant **Kimberly C. Claeys, PharmD**, Abbvie: Advisor/Consultant|bioMérieux Inc.: Advisor/Consultant|bioMérieux Inc.: Speaker|La Jolla Pharmaceuticals: Advisor/Consultant|Melinta Therapeutics: Advisor/Consultant **Jason C. Gallagher, PharmD**, Entasis: Advisor/Consultant|Merck: Advisor/Consultant|Merck: Grant/Research Support|Qpex: Advisor/Consultant|Shionogi: Advisor/Consultant|Spero: Advisor/Consultant **Emily L. Heil, PharmD, MS**, Wolters Kluwer-LexiComp: Advisor/Consultant **Wesley D. Kufel, PharmD, BCPS, BCIDP, AAHIVP**, Merck and Co: Grant/Research Support **Amy Mathers, MD, D(ABMM)**, Merck: Advisor/Consultant **Erin K. McCreary, PharmD**, Abbvie: Advisor/Consultant|Ferring: Advisor/Consultant|GSK: Honoraria|La Jolla (Entasis): Advisor/Consultant|LabSimply: Advisor/Consultant|Merck: Advisor/Consultant|Shionogi: Advisor/Consultant|Shionogi: Honoraria **Christopher Polk, MD**, ViiVHealthcare: Job change to work for ViiV as Medical Director **Michael J. Satlin, MD**, AbbVie: IDMC member|Biomerieux: Grant/Research Support|Merck: Grant/Research Support|SNIPRBiome: Grant/Research Support **Michael Veve, PharmD, MPH**, National Institutes of Health: Grant/Research Support|Paratek Pharmaceuticals: Grant/Research Support **jason M. Pogue, PharmD**, AbbVie: Advisor/Consultant|Entasis: Advisor/Consultant|Ferring: Advisor/Consultant|GSK: Advisor/Consultant|Merck: Advisor/Consultant|Merck: Grant/Research Support|Qpex: Advisor/Consultant|Shionogi: Advisor/Consultant

